# Service innovation management practices in the telecommunications industry: what does cross country analysis reveal?

**DOI:** 10.1186/s40064-015-1580-8

**Published:** 2015-12-23

**Authors:** Syed Abidur Rahman, Seyedeh Khadijeh Taghizadeh, T. Ramayah, Noor Hazlina Ahmad

**Affiliations:** Department of Business Administration, Stamford University Bangladesh, 744, Saat Masjid Road, Dhaka, Bangladesh; School of Management, Universiti Sains Malaysia, Pulau Penang, Malaysia

**Keywords:** Service innovation practices, Innovation process, Cross-functional organisation, Tools/technology, Telecommunications industry, Malaysia, Bangladesh

## Abstract

Service innovation management practice is currently being widely scrutinized mainly in the developed countries, where it has been initiated. The current study attempts to propose a framework and empirically validate and explain the service innovation practices for successful performance in the telecommunications industry of two developing countries, Malaysia and Bangladesh. The research framework proposes relationships among organisational culture, operating core (innovation process, cross-functional organisation, and implementation of tools/technology), competition-informed pricing, and performance. A total of 176 usable data from both countries are analysed for the purpose of the research. The findings show that organisational culture tends to be more influential on innovation process and cross-functional organisation in Malaysian telecommunication industry. In contrast, implementation of tools/technology plays a more instrumental role in competition-informed pricing practices in Bangladesh. This study revealed few differences in the innovation management practices between two developing countries. The findings have strategic implications for the service sectors in both the developing countries regarding implementation of innovative enterprises, especially in Bangladesh where innovation is the basis for survival. Testing the innovation management practices in the developing countries perhaps contains uniqueness in the field of innovation management.

## Background

Innovation coupled with performance of firms is a subject with significant attention within academia (Damanpour 2014) due to its rapid and dramatic impact on society and organisations across borders. In order to achieve ultimate goal in an organisation, managerial practices and activities can play a vital role. In this regards, few rudimentary and imperative management practices is considered in this study context to understand to what extent such practices contribute organisations for accomplishing the performance specifically in developing context. Scholars claim that countries and regions are endowed with diverse types of resources and infrastructures (Chen and Hsiao 2013) which rely on their own organisational culture how to practice (Aycan et al. 2000). Earlier literatures illustrated the influence of national and organisational culture on different managerial practices in the organisations (Ardichvili et al. 2006) as well as on successful innovation (Lee et al. 2013; Büschgens et al. 2013). In the context of ‘culture’ issue, some of the scholars have asserted that national culture has an influence along with other spectrums on the organisation and its culture (Tayeb 1994). To be more specific, literatures suggest that organisational culture is the integral part of the national culture (Iorgulescu and Marcu 2015). However, Hogan and Coote (2014) noted that despite much focused attention on the topic of organisational culture and innovation, the extant literature does not sufficiently document the organisational culture that enables innovation. To have successful innovation, scholars gave importance to three operating core of innovation as fundamental aspects of innovation management. These three operating core are innovation process, cross-functional organisation, and implementation of tools/technology introduced by Hull et al. (1996). These practices facilitate service companies in managing their new service development process in a best way (Collins and Hull 2002) as it is proved to be faster, cheaper, and better for service development than serial alternatives (Liker et al. 1999). As scholars highlighted, innovation process, cross-functional organisation, and implementation of tools/technology are increasingly necessary for survival under conditions of hyper competition (Hull 2004). Further, literatures suggest that in the process, a great deal of effort must be put in the implementation of new products/services (Orfila-Sintes et al. 2005). Innovation process considers various activities include effectiveness in market assessment, bench marketing, identify customer needs, quality function, and review on the design of the products (Hull 2004). This guidance can create value for customers who are the focus of innovation (De Jong and Vermeulen 2003). In addition, cross-functional teams are often seen as key for innovation projects (Blindenbach-Driessen 2015) which carries out every practice and process in a systematic and sustainable way (Weiss and Legrand 2011). It is generally an accepted notion that people are of central importance in cross-functional organisation as each has capabilities to find and solve problems. Cross-functional organisation with high performance teamwork can bring success to firms, while without could be a reverse situation (Weiss and Legrand 2011). In the stream of innovation literature, tools/technology mainly represents the usage of computer and information technology (CIT). Most service firms are knowledge-based and heavily depend on information technology (IT) (Hull and Tidd 2003b), hence, IT can facilitate the decision making process in the development cycle in a shorter time (Hull 2004). In addition, CIT enables team members to share their experience in service development cycle and systematically compare their services with competitors (Tidd and Bessant 2009). It allows management to evaluate and control all the projects through stored day-to-day information as well to learn and conduct staff training upon reviewing customer and user satisfaction, evaluating projects, and audits (Mudrak et al. 2005).

Moreover, this study has considered competition-informed pricing as important practices for new service development. Competition-informed pricing refers to the prices of competing product that are used as a benchmark instead of customer demand. The competition-informed pricing assumes that the cost structure of the company would be such a way that matches with the competitors’ pricing (Shapiro and Jackson 1978). According to Hinterhuber (2004), while making the pricing decisions the manager must take into consideration the competitive perspective which facilitates to inform the competitors’ pricing. The purpose of choosing competition-informed pricing is due to the selection of telecommunications industry in the current context. Competition-informed pricing in the telecommunications industry plays persuading role. It is matter of fact that in the telecommunications industry, the level of competition is more intense compared to any other industry, irrespective of a country’s economic and social state. The market structure of telecommunications industry is considered as oligopoly. In the oligopoly market, there are only few firms which have considerable control over their prices, but each firm must consider the course of actions, activities, and reactions of the rivals (Noam 2006). Hence, an organisation cannot overlook the importance of today’s hyper competitive market in their innovation process because researchers noted that innovation has a synchronized relationship with competitors (Goto 2009).

Finally, the study has attempted to reveal the impact of such practices on the innovation performance. Performance reflects the business initiatives and strategies taken by the firm. Previous researchers argued that innovation in an organisation directly and positively influences the improvement in business performance (Tidd et al. 2005). Innovation as a firm’s unique resource can lead to competitive advantage and improvement in performance, effectiveness, and efficiency (Barney 1991). If firms are highly focused on innovation, they will be more successful in the offering of new products and services where subsequently it will result in greater performance (Eisingerich et al. 2009). However, over the past years many of countries face difficulties in strengthening innovation performance (OECD 2007) which diverges due to the capacity to innovate. To do so, the study has framed this research in the telecommunication industry of two countries. Most importantly, the study intended to test a framework in developing countries which has partially been molded and tested in developed countries. As in the recent literatures, scholars have solicited to modify and test management theories and framework in emerging economies which are typically built in the northern part of the globe (Barrett et al. 2015). However, this is a prospect to substantiate whether framework initiated in the developed countries explicate similar underlined causal effects across developing countries. We have chosen two Asian countries, one of which is considered as innovation driven country (Malaysia), and another considered as only factor driven country with insufficient capacity to innovate (Bangladesh) (World Economic Forum 2015). Bangladesh is one of the prominent member of the world “Next Eleven” group which is considered the most lucrative emerging economy group amongst others in the globe and the country is planning to step in the middle income country by the year of 2021 (Planning Commission 2012). On the other hand, Malaysia is the one of the most potential developing countries which plans to enter the club of ‘developed countries’ by the year 2021 (Malaysian Investment Development Authority 2014). However, to achieve such economical shift by the year of 2021, it is presumed that innovation and its practices in the industries can be one of the driving forces.

In both the countries, telecommunications industry plays leading role in the development of the economy. Profile of the telecommunications industry indicates a proximate similarity in terms of operations and ownership. DiGi a Malaysian telecommunications company and GrameenPhone a Bangladeshi telecommunications company are both a foreign subsidiary of Telenor group, Norway. DiGi holds the second position in terms of market share in Malaysia and GrameenPhone holds the largest market share in Bangladesh. On the other hand, Robi Axiata, a Malaysian subsidiary of the Celcom Axiata group, is operating in Bangladesh with significant market share in the country as well as in Malaysia.

Therefore, the current study attempts to propose a framework and empirically validate and explain the service innovation practices between the emerging countries as researchers suggested limited study in these context _ENREF_44 (Taghizadeh et al. 2014). The result may contribute for the policy maker as guideline to enhance the innovation performance through firm resources and capabilities. This paper is structured in seven sections. The second section provides an overview of the theoretical justification of the variables that help the reader to understand the proposed research framework as well as hypotheses formulation. The research methodology and the findings of the empirical analysis used in the study are discussed in section three. In section four, a discussion derived from the result is presented. Implication, conclusion, and limitation with future direction of the research are presented in section five, six, and seven, respectively.

## Theoretical background and hypothesis development

Todays, changes are taking place everywhere, which raising complexity among the environment e.g. changes in economic condition lead to the opening of new markets, while closing others (van Riel 2005). Such a domino effect subsequently increases the level of global competition and rivalry among the companies (van Riel 2005). To overcome the complexity, management need to have a balanced, comprehensive, and proactive approach (Ottenbacher 2007). Scholars believe that successful service innovation not only depends on how a firm manages projects, coordinates imputes of different functions, and links up with its customer, but also relies on being able to develop strategic approaches and look widely (Tidd et al. 2005). Literature on new service development reveals that the growth and performance of any organisation rely on an efficient management of innovation in a competitive climate (Jiménez-Jiménez and Sanz-Valle 2011; Tidd and Bessant 2009).

In the literature, a composite model was illustrated comprising of three managerial practices: innovation process, cross-functional organisation, and implementation of tools/technology introduced by Hull et al. (1996). These practices facilitate service companies in managing their new service development process in a best way (Collins and Hull 2002) as it is proved to be faster, cheaper, and better for service development than serial alternatives (Liker et al. 1999). Innovation process, cross-functional organisation, and implementation of tools/technology are known as the operating core and are increasingly necessary for survival under conditions of hyper competition (Hull 2004). In this operating core both marketing and developmental operations are included in contrast to literature dealing with the market on the one hand and organisation behaviour on the other (Hull 2004).

Innovation process represents a disciplined practice in order to control the procedure from idea generation to launch (Hull and Tidd 2003a). According to Hull and Tidd (2003a) and Liker et al. (1999), innovation process denotes the mechanistic form of an organisation where rules and regulations are structured and maintained accordingly. Hull and Tidd (2003a) pointed out that in the setting of innovative process, organisations tend to be effective, efficient, and characterized by standardized procedures. A clear division of labour and an authoritarian chain of command prevail, while the companies embrace the innovative process for the service innovation management (Liker et al. 1999).

Cross-functional organisation involves the coordination of people at all the stages of innovation practices (Tidd et al. 2005). Liker et al. (1999) asserted an innovative organisation is characterised by an organic setting that tends to be flexible and characterised by few rules and standard procedures. Teamwork and a creative combination of various views, perspectives and disciplines recognizes innovative organisational practices (Tidd and Bessant 2009). Co-involvement of operations people, who are developing the services and delivering systems support behind the scenes, is necessary for firms success (Magnusson et al. 2003).

Tools/technology denotes enabling computer information technologies (CIT) in supporting communication (Hull et al. 1996). According to Collins and Hull (2002), organisational transformation and transaction capabilities are enhanced by the adoption of CIT’s tools, such communication devices and data distribution approaches. As the complexity of the business environment has been increased, it requires organisations to have a collaborative and creative working place through the implementing of CIT’s tools (Klein and Dologite 2000). According to scholars, the proliferation of information and technology has created a revolution in the current trend with a wider economic perspective across national borders (Erumban and De Jong 2006).

However, Hull and Tidd (2003a) found that training and championing ‘as a part of organisational culture’ influence on shaping up the innovation process, cross-functional organisation, and implementation of tools/technology of service-oriented companies. Hence, we propose that organisational culture can play a stimulus role in practicing the operating core. Scholars noted that organisational culture plays an influential role in the management practices of the firms (Zammuto and O’Connor 1992). Organisational culture is a complex set of values, beliefs, assumptions, and symbols that a firm should institute in its business operation (Miron et al. 2004; Chang and Lin 2007; Barney 1986; Martins and Terblanche 2003). According to Naranjo-Valencia et al. (2011), to facilitate the implication of innovation successfully, organisations should meet the requirements of internal behaviour and external relations which comply with the organisational culture. In fact, organisational culture is a source of new ideas within the organisation (Uzkurt et al. 2013). As suggested by Chang and Lin (2007), this paper conceptualizes organisational culture by considering the four cultural traits (i.e. cooperativeness, innovativeness, consistency, and effectiveness) into a single domain. Cooperativeness focuses primarily on cooperation to each other as extended family which represents a strong team work and trust to each other. Innovativeness can be characterized with a focus on creativity, adaptability, and dynamism which allows the employees for the self-development. Consistency emphasizes on maintaining order, rules and regulations, uniformity, and efficiency throughout the organisational structure. The cultural trait of effectiveness indicates the competitiveness, goal achievement, and efficiency of the organisational activities. Therefore, this paper proposes that organisational culture may have effect on the operating core and thus the following hypotheses would be worthy of testing:

**H1.** Organisational culture facilitates the practise of continuous process improvement in service development.

**H2.** Organisational culture enables the practise of cross-functional organisation to a great level in service development.

**H3.** Organisational culture accelerates the implementation of information technology tools in service development.

In oligopoly market high barriers to entry for new competitors exist to a greater extent. Such barriers to entry impede the other new entrants in competing in the market due to the high start-up capital cost (McConnell et al. 2009). To achieve a desire performance in oligopoly market, each firm must consider the course of actions, activities, and reactions of the rivals (Noam 2006). So, competition-informed pricing as how to set prices using information gathered from competitors can be helpful in order to deal with pricing complexity. Hinterhuber (2004) believes that while making pricing decisions a manager must take into consideration the competitive perspective. Competition-informed pricing has the tendency to enhance the likelihood of setting the right price by a competitor’s innovation practices, including pricing that may match or exceed the firms’ price for innovated products and services. The price of competitive products and competitive advantages of competitors dictate that the firm needs to make an evaluation on the firms’ position in the market vis-a-vis the competitors (Ingenbleek et al. 2003). The competitor’s current price strategy and strength to react are important components for competition-informed pricing. While firms practice competition-informed pricing, it is also imperative for them to consider the market structure, degree of competition in the market, and the competitive advantages of competitors in the market. Such activities in fact refer to the overall knowledge of the competition by the market players. In this vein, this research suggests that the operating core can facilitate the efforts of managers to gather information related to competitors. For example, process involves external investigation for developing new products and services (Hull 2003). It may help firms in the practice of price decision making through involvement of the functional departments in the procedure towards understanding the strategic movement of rivals in the market. Inter-functional coordination and cooperation are deemed instrumental in efficient innovation management in gathering data regarding the right price from the perspectives of competitors. It can be assumed that the degree of competition can be understood through the propensity of coordination of people in an organisation. Or else, CIT’ tools along with continuous updating of the service development process may facilitate firms in gathering competitors’ price related information in a shorter time. Considering the above discussion, the following hypothesis is formulated:

**H4.** Continuous process improvement increases the level of gathering competition-informed pricing in service development.

**H5.** A cross-functional organisation facilitates the level of gathering competition-informed pricing in service development.

**H6.** Implementation of information technology tools for gathering competition-informed pricing is easier in service development.

Previous study found the relationship between competition-informed pricing firms performance (Ingenbleek et al. 2003). In fact it is difficult to find an ideal measurement for business performance particularly in collecting performance data. In the past studies, the performance of an organisation is frequently evaluated by the simple outcomes of financial indicators such as return on investment (ROI), return on sales, or sales growth. This study measured non-financial performance focusing on innovation activities in terms of new service development and delivery process improvement which has been also found in the earlier research (e.g. Hull 2003; Hull and Tidd 2003a). Coming up with upgraded features, higher quality of services, shorter time for delivery of services, reducing cost of service development, higher quality in the delivery process are the major indicators to measure the performance of the organisation in terms of new service development and delivery process (Hull and Tidd 2003a). To achieve better performance, it is expected to implement appropriate pricing practice (Hultink et al. 1997) as scholars have also asserted that setting a right price drives superior performance for firms (Dutta et al. 2003). Competition-informed pricing increases the chance of setting the right price by knowing competitor’s innovation (Ingenbleek et al. 2003). Gathering information from competitors’ price strategy enable a quantitative evaluation of the firm’s relative position (Ingenbleek et al. 2003). Therefore, we propose that understanding the competitors’ trend of pricing, degree of competition, and market structure will enable service companies to upgrade services with new features and reduce the time of response. Thus, the following hypothesis is presented for testing:

**H7.** The greater the practice of competition-informed pricing, the higher the level of performance improvement.

Based on the above discussion, this paper proposes that competition-informed pricing can mediate the relationship between operating core and performance. There is hardly any research being conducted to examine the impact of the operating core on performance of service firms through the possible role of competition-informed pricing. The rationale for testing this mediating effect arises from the market structure of telecommunications industry. Practicing operating core of the service innovation perhaps is not enough to achieve the performance enhancement of service industries. While service-based companies embrace the operating core of innovation practices, they subsequently need to understand the position of their competitors in the market. It is a generally accepted notion that in a competitive market, each and every company follows the competitors’ pricing and pricing strategy. Vermeulen and van der Aa (2003) mentioned that most organisations use services which are developed by some competitor in order to adjust the competitors’ product in their innovation process. To a greater extent, such companies try to get as much information on the competitors’ price. By understanding the pricing position of competitors, service companies attempt to attain higher performance. Thus, the following hypotheses would be worth of testing:

**H8.** Competition-informed pricing mediates the relationship between process and performance.

**H9.** Competition-informed pricing mediates the relationship between organisation and performance.

**H10.** Competition-informed pricing mediates the relationship between implementing tools/technology and performance.

After all, we believe that culture, service innovation practice, pricing, and firm’s performance of mobile phone companies should differ significantly between Malaysia and Bangladesh. The reason for choosing these two contexts is discussed in introduction part. Therefore, we test all path relationships though multi group analysis.

**H11**: All the hypothesised relationships in the proposed framework will differ between Malaysia and Bangladesh telecommunication companies.

**Fig. 1:**
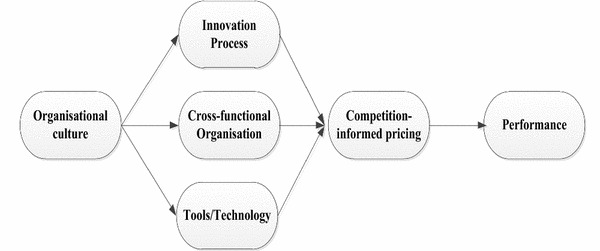
Research framework

Thus, the research framework (Fig. 1) aims to explore the relationship of organisational culture as a predictor of operating core (innovation process, cross-functional organisation, and implementation of tools/technology) for new service development. Further, we draw attention to explore the mediating role of competition-informed pricing practices between the relationship of operating core practices and performance.

## Research methodology and result

### Sample and data

To test the research framework and hypotheses, we considered telecommunications industry in Bangladesh and Malaysia. In Bangladesh, out of six, three top largest telecommunications companies (GrameenPhone, Robi Axiata, and Airtel) were chosen as they contain more than 60 % of the total market share in the country. Similarly, three top largest telecommunications companies from Malaysia (DiGi, Maxis, and Celcom Axiata) were chosen out of six, which are holding more than 60 % of the total market share in the country as well. The purposive sampling was chosen because specific managers form the respondent pool for the research questionnaire survey. In Malaysia, there are 820 branch offices for the DiGi, Maxis, and Celcom that we could collect 98 usable data. In Bangladesh, there are in total 621 branch offices and the usable collected data is 78. To run the analysis of the current framework with three predictors, it is required to have a minimum sample size of 77, which would generate a power of 0.80 for a model with medium effect size (Hair et al. 2013). Therefore, a total of 176 usable data from both countries are analysed for the purpose of the research. Table 1 provides the demographic statistics of the sample data.

**Table 1:** Demographic profile of respondent

	Full sample (N = 176)		Bangladesh (N = 78)		Malaysia (N = 98)	
	Percent	Cumulative percent	Percent	Cumulative percent	Percent	Cumulative percent
Job title						
Assistant Manager	33.0	33.0	28.2	28.2	36.7	36.7
Deputy Manager	12.5	45.5	21.8	50.0	5.1	41.8
General Manager	10.8	56.3	11.5	61.5	10.2	52.0
Key Account Manager	4.0	60.2	5.1	66.7	3.1	55.1
Manager	39.8	100.0	33.3	100.0	44.9	100.0
Department						
Business Operation	2.8	2.8	1.3	1.3	4.1	4.1
Customer Service Department	17.0	19.9	21.8	23.1	13.3	17.3
Human Resource Department	3.4	23.3	6.4	29.5	1.0	18.4
IT	4.5	27.8	0.0	0.0	8.2	26.5
Marketing	40.3	68.2	39.7	69.2	40.8	67.3
Product Development	7.4	75.6	10.3	79.5	5.1	72.4
Sales and Service	24.4	100.0	20.5	100.0	27.6	100.0
Experience in telecommunication industry						
5 years or less	23.9	23.9	23.1	23.1	24.5	24.5
6–8 years	42.0	65.9	46.2	69.2	38.8	63.3
9–11 years	17.0	83.0	20.5	89.7	14.3	77.6
12 years or more	17.0	100.0	10.3	100.0	22.4	100.0
Experience with the current company						
5 years or less	51.7	51.7	56.4	56.4	48.0	48.0
6–8 years	27.3	79.0	29.5	85.9	25.5	73.5
9–11 years	9.7	88.6	10.3	96.2	9.2	82.7
12 years or more	11.4	100.0	3.8	100.0	17.3	100.0

The questionnaire was developed from past studies. The items for organisational culture (OC1 to OC9) were taken from Chang and Lin (2007). In the survey questionnaire, the respondents were asked to respond on the items of organisation culture on 5-point Likert scale (1 = strongly disagree to 5 = strongly agree) with the question “*How much do you agree on the following practices…?.*”

The items for innovation process (PRC1–PRC5), cross-functional organisation (ORG1–ORG5), and tools/technology (TLS1–TLS5) were taken from Hull (2003) and Hull and Tidd (2003a), and anchored on 5-point Likert scale (1 = very low extent to 5 = very high extent).

While measuring the innovation process, the respondents were asked to rate the items considering the following statement, “*By the practice of innovation process, our company is…*”

To measure the cross-functional organisation the statement was “*By the practice of cross*-*functional organisation, our company has…*”

Tools/technology was measured on the basis of following statement “*In the implementation of information technology tools, our company has…*”

The items for competition-informed pricing (COMIP1–COMIP5) were taken from Ingenbleek et al. (2003), and measured on 5-point Likert scale (1 = very low extent to 5 = very high extent). The managers were asked to indicate “*To what extent your company take into consideration….?.*”

The items for performance were taken from Hull and Tidd (2003a) in terms of service development (SD1–SD5) and delivery process (DP1–DP5) measured on 5-point Likert scale (1 = very low extent to 5 = very high extent). While measuring the performance, the respondents were asked to rate the items considering the following statement “*To what extent has your operation system changed based on the following…*” Details of the items have been illustrated in “Appendix”.

### Data analysis

To ensure that there is no Common Method bias in the questionnaire survey, we performed Harman’s single factor test. This revealed that the first factor accounted for 45.018 % of variance, which is less than threshold level of 50 % of total variance explained (Podsakoff et al. 2003).

In this study, to see whether there any differences between subsidiaries group exist (DiGi in Malaysia and GrameenPhone in Bangladesh are both subsidiaries of Telnor group; Robi in Bangladesh and Celcom in Malaysia are subsidiaries of Axiata group), an independent-sample *t* test was conducted to compare the six variables. Parent companies Telenor and Axiata were considered as two groups, where, DiGi and GrameenPhone were considered as group 1 and Celcom and Robi were grouped as 2. The results show that the p value from the independent t test for five variables is not significant except for one variable that is organisational culture. Organisational culture shows some slight difference in the means between the two groups of subsidiaries. Therefore, the effect size test was calculated to determine the magnitude of the difference as suggested by (Cohen 1988). The effect size is determined by the Cohen’s d value. The formula to get the Cohen’s d is:

$${\text{Cohen's d}} = {\text{difference between sample mean}}/{\text{pooled standard deviation}}$$

The interpretation for effect size using Cohen’s d test value belonging to the categories: 0.20–0.49 (small), 0.50–0.79 (medium), and above or equal to 0.80 (large). The result of the test indicates that the effect size of the variable is small (0.21), therefore, the homogeneity of two groups of subsidiaries is established. The small effect size indicates that the response bias is not a threat.

In order to achieve our research objectives and analyse the measurement and structural model, we considered the structural equation model (SEM) with PLS approach, specifically the SmartPLS version 2.0 M3 Beta (Ringle and Wende 2005). PLS-SEM can be viewed as quite similar to multiple regression analysis to examine possible relationships with less emphasis on the measurement model (Hair et al. 2013). The individual path coefficients in the PLS structural model can also be interpreted as standardised beta coefficients of ordinary least square regression (Götz et al. 2010). Each path coefficient’s significance can be accessed through a bootstrapping procedure where significant paths showing the hypothesised direction empirically support the proposed causal relationship and vice versa (Hair et al. 2011; Yung and Bentler 1994; Efron 1979). Bootstrapping in PLS is a nonparametric test which involves repeated random sampling with replacement from the original sample to create a bootstrap sample and to obtain standard errors for hypothesis testing (Hair et al. 2011). Regarding the number of re-sampling, Chin (2010) suggested to perform bootstrapping with 1000 re-samples. In the current study, the bootstrapping procedure with 1000 re-samples was used to test the significance of the path coefficients (regression coefficients). The path coefficients have standardized values between −1 and +1. The estimated path coefficients close to +1 represents a strong positive linear relationship and vice versa for negative values (Hair et al. 2013). In addition, to carry out a multi-group analysis between the companies of the two countries, PLS is considered to be more appropriate to explore the differences between them. The respondents of Bangladesh telecommunications sector’s managers and Malaysian telecommunications sector’s managers were split into two data sets (Bangladesh = 78 samples and Malaysia = 98 samples). To estimate the structure model, all criteria such as convergent validity, discriminant validity, and measurement invariance were checked separately as suggested by Hair et al. (2013).

Factor loadings of the items, average variance extracted (AVE), and composite reliability (CR) are used to assess convergence validity of the data (Hair et al. 2009). To ensure the indicators’ reliability, the main loading and cross-loading of items are checked. In accordance with Chin (1998), we retained the items which exceeded the recommended value of 0.6 while three items (OC8, OC9, TLS4) were found to be below the cut off value were deleted. Two items (OC4 and ORG5) were deleted because of cross-loading. The AVE of all the constructs exceeded the cut off value of 0.5 suggested by in literature (Henseler et al. 2009; Hair et al. 2013). The CR values of the constructs were found to have a minimum threshold of 0.7 suggested by Hair et al. (2011). Table 2 shows the results.

**Table 2:** PLS factor loadings, CR, and AVE of full and country samples

Constructs	Items	Full sample (n = 176)			Malaysia (n = 98)			Bangladesh (n = 78)		
		Loading	AVE	CR	Loading	AVE	CR	Loading	AVE	CR
Organisation culture	OC1	0.851	0.728	0.941	0.739	0.661	0.921	0.921	0.753	0.948
	OC2	0.845			0.792			0.829		
	OC3	0.901			0.890			0.886		
	OC5	0.844			0.822			0.821		
	OC6	0.814			0.798			0.855		
	OC7	0.861			0.831			0.890		
Innovation process	PRC1	0.759	0.668	0.910	0.717	0.653	0.904	0.770	0.573	0.870
	PRC2	0.791			0.775			0.667		
	PRC3	0.840			0.855			0.771		
	PRC4	0.846			0.841			0.803		
	PRC5	0.847			0.844			0.767		
Cross-functional organisation	ORG1	0.867	0.719	0.911	0.897	0.740	0.919	0.763	0.590	0.851
	ORG2	0.852			0.877			0.762		
	ORG3	0.879			0.870			0.844		
	ORG4	0.791			0.792			0.696		
Tools/technology	TLS1	0.826	0.715	0.909	0.821	0.705	0.904	0.785	0.660	0.885
	TLS2	0.922			0.934			0.878		
	TLS3	0.840			0.886			0.771		
	TLS5	0.787			0.698			0.810		
Competition-informed pricing	COMIP1	0.815	0.743	0.935	0.862	0.815	0.957	0.713	0.595	0.880
	COMIP2	0.877			0.897			0.826		
	COMIP3	0.870			0.906			0.773		
	COMIP4	0.893			0.944			0.795		
	COMIP5	0.854			0.903			0.746		
Performance	DP1	0.823	0.672	0.953	0.806	0.653	0.950	0.817	0.655	0.950
	DP2	0.811			0.840			0.750		
	DP3	0.832			0.853			0.796		
	DP4	0.801			0.798			0.789		
	DP5	0.814			0.823			0.764		
	SD1	0.797			0.760			0.822		
	SD2	0.824			0.819			0.808		
	SD3	0.842			0.836			0.841		
	SD4	0.823			0.745			0.868		
	SD5	0.830			0.795			0.830		

Items OC4, OC8, OC9, ORG5, and TLS4 were deleted

*CR* composite reliability, *AVE* average variance extracted

After convergent validity, we analysed the discriminant validity of the model. The discriminant validity was assessed for both the full and split sample by comparing the correlations between constructs and the square root of the average variance extracted for that construct (Fornell and Larcker 1981). The results show that the square roots of AVEs are greater in all cases than the off-diagonal elements in their corresponding row and column, suggesting that the required discriminant validity was achieved (Table 3). In total, the measurement model demonstrated adequate convergent validity and discriminant validity.

**Table 3:** Discriminant validity of data sets

	1	2	3	4	5	6
Full sample						
1. COMIP	*0.862*					
2. OC	0.543	*0.853*				
3. Cross-functional organisation	0.550	0.584	*0.848*			
4. Performance	0.602	0.651	0.561	*0.820*		
5. Innovation process	0.534	0.520	0.703	0.532	*0.817*	
6. Tools/technology	0.546	0.567	0.558	0.587	0.600	*0.845*
Malaysia						
1. COMIP	*0.903*					
2. OC	0.508	*0.813*				
3. Cross-functional organisation	0.477	0.651	*0.860*			
4. Performance	0.562	0.557	0.535	*0.808*		
5. Innovation process	0.493	0.545	0.648	0.579	*0.808*	
6. Tools/technology	0.400	0.471	0.443	0.480	0.512	*0.839*
Bangladesh						
1. COMIP	*0.771*					
2. OC	0.505	*0.868*				
3. Cross-functional organisation	0.552	0.350	*0.768*			
4. Performance	0.596	0.659	0.457	*0.809*		
5. Innovation process	0.456	0.314	0.639	0.326	*0.757*	
6. Tools/technology	0.676	0.545	0.530	0.623	0.544	*0.812*

Diagonal elements are the square root of the AVE of the reflective scales while the diagonals are the correlations between constructs

*COMIP* competition-informed pricing, *OC* organisational culture

Measurement invariance was tested. According to Hair et al. (2013), researchers should ensure the construct measures are invariant across the groups while comparing path coefficients across the groups using the PLS-MGA parametric. Bootstrapping is used according to the number of the observation in the data set separately for each group. Through outer weights and standard errors for each group and using the Levene’s test suggested by Hair et al. (2013), the invariance test is checked for all items. In this test, if the test for equality of group variance is significant, then the unequal standard errors are assumed and the test statistic (*t* value) is computed as follows:

$$S_{1 2} = \sqrt {S_{1}^{2} + S_{2}^{2} }$$

If the test for equality of group variance is not significant, equal standard errors are assumed and the test statistic (*t* value) is computed as follows:

$$S_{1 2} = \left( {\sqrt {\frac{{\left( {N_{1} - 1} \right)^{2} }}{{\left( {N_{1} + N_{2} - 2} \right)}} \,. \,S_{1}^{2} + \frac{{\left( {N_{2} - 1} \right)^{2} }}{{\left( {N_{1} + N_{2} - 2} \right)}} \cdot S_{2}^{2} } } \right) \cdot \left( {\sqrt {\frac{1}{{N_{1} }} + \frac{1}{{N_{2} }}} } \right)$$

The criterion is that at least two items should not differ in the measurement items of each construct. The result shows that the there is no significant difference among the two groups. Table 4 shows the results.

**Table 4:** Invariance test

	Bangladesh		Malaysia		Test for equality of variance	t value	p	Sig.
	Beta	SE	Beta	SE				
OC1 ← OC	0.197	0.016	0.144	0.033	1.000	1.325	0.187	–
OC2 ← OC	0.154	0.035	0.190	0.020	0.000	0.899	0.370	–
OC3 ← OC	0.229	0.036	0.222	0.016	0.000	0.177	0.860	–
OC5 ← OC	0.183	0.031	0.221	0.020	0.001	1.050	0.296	–
OC6 ← OC	0.221	0.034	0.213	0.023	0.004	0.183	0.855	–
OC7 ← OC	0.168	0.030	0.233	0.025	0.253	1.683	0.094	*
PRC1 ← Process	0.301	0.070	0.213	0.031	0.000	1.149	0.253	–
PRC2 ← Process	0.162	0.066	0.194	0.032	0.000	0.433	0.666	–
PRC3 ← Process	0.279	0.065	0.263	0.023	0.000	0.234	0.816	–
PRC4 ← Process	0.346	0.060	0.255	0.026	0.000	1.414	0.160	–
PRC5 ← Process	0.218	0.061	0.306	0.031	0.000	1.286	0.201	–
ORG1 ← Organisation	0.295	0.051	0.357	0.031	0.000	1.045	0.298	–
ORG2 ← Organisation	0.306	0.053	0.254	0.026	0.000	0.876	0.383	–
ORG3 ← Organisation	0.381	0.041	0.289	0.025	0.000	1.919	0.057	*
ORG4 ← Organisation	0.317	0.065	0.259	0.029	0.000	0.832	0.407	–
TLS1 ← Tools/technology	0.253	0.048	0.305	0.051	0.953	0.736	0.463	–
TLS2 ← Tools/technology	0.314	0.033	0.339	0.034	0.902	0.518	0.605	–
TLS3 ← Tools/technology	0.309	0.038	0.290	0.031	0.223	0.381	0.704	–
TLS5 ← Tools/technology	0.355	0.041	0.251	0.052	0.999	1.514	0.132	–
COMIP1 ← COMIP	0.213	0.029	0.185	0.020	0.008	0.795	0.428	–
COMIP2 ← COMIP	0.298	0.028	0.232	0.019	0.004	1.958	0.052	*
COMIP3 ← COMIP	0.231	0.029	0.201	0.015	0.000	0.928	0.356	–
COMIP4 ← COMIP	0.287	0.031	0.246	0.015	0.000	1.209	0.229	–
COMIP5 ← COMIP	0.262	0.029	0.241	0.018	0.000	0.592	0.555	–
DP1 ← Performance	0.108	0.017	0.118	0.015	0.461	0.466	0.642	–
DP2 ← Performance	0.108	0.021	0.121	0.015	0.032	0.515	0.607	–
DP3 ← Performance	0.149	0.023	0.125	0.017	0.046	0.857	0.393	–
DP4 ← Performance	0.106	0.016	0.155	0.022	1.000	1.695	0.092	*
DP5 ← Performance	0.134	0.022	0.153	0.020	0.534	0.638	0.524	–
SD1 ← Performance	0.127	0.022	0.102	0.020	0.556	0.842	0.401	–
SD2 ← Performance	0.100	0.017	0.109	0.018	0.970	0.339	0.735	–
SD3 ← Performance	0.147	0.020	0.145	0.021	0.920	0.087	0.930	–
SD4 ← Performance	0.130	0.018	0.108	0.027	1.000	0.651	0.516	–
SD5 ← Performance	0.127	0.020	0.101	0.019	0.765	0.954	0.341	–

**Fig. 2:**
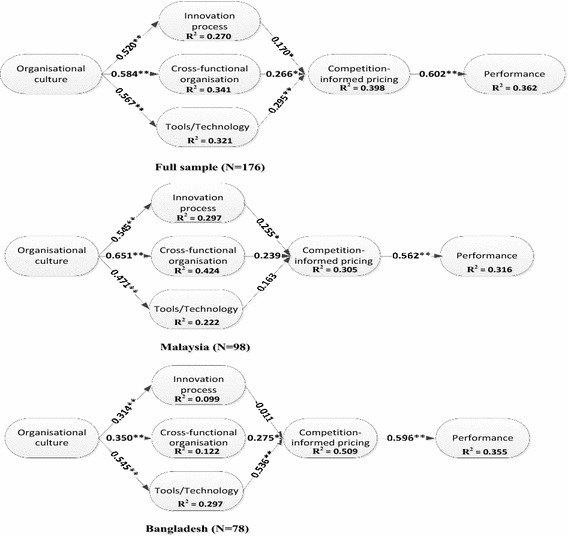
Structural models. **p < 0.01, *p < 0.05

After testing measuring model, the structural model has been analysed. The *R*^2^ and the path coefficients (beta and significance) show how well the data supported the hypothesized model (Chin 1998). We used the bootstrapping method with a resampling of 1000 to estimate the significance of the path coefficients (Chin 1998). The path coefficients for full and split data are shown in Table 5 and Fig. 2.

**Table 5:** Result for direct relationships

	Path	Full sample (n = 176)				Malaysia sample (n = 98)				Bangladesh sample (n = 78)			
		Std. Beta	SE	t value	Result	Std. Beta	SE	t value	Result	Std. Beta	SE	t value	Result
H1	OC → Process	0.520	0.055	9.370**	S	0.545	0.069	7.925**	S	0.314	0.088	3.582**	S
H2.	OC → Organisation	0.584	0.058	10.06**	S	0.651	0.060	10.94**	S	0.350	0.082	4.269**	S
H3.	OC → Tools/technology	0.567	0.051	11.20**	S	0.471	0.091	5.195**	S	0.545	0.064	8.584**	S
H4.	Process → COMIP	0.170	0.103	1.649*	S	0.255	0.138	1.842*	S	−0.011	0.126	0.086	NS
H5.	Organisation → COMIP	0.266	0.119	2.234*	S	0.239	0.151	1.585	NS	0.275	0.144	1.916*	S
H6.	Tools/technology → COMIP	0.295	0.087	3.375**	S	0.163	0.112	1.455	NS	0.536	0.100	5.347**	S
H7.	COMIP → Performance	0.602	0.044	13.83**	S	0.562	0.056	10.07**	S	0.596	0.069	8.660**	S

*COMIP* competition-informed pricing, *OC* organisational culture

** p < 0.01, * p < 0.05

*Hypotheses related to organisational culture and operating core* From the analysis, we found H1 was supported in the full data (β = 0.520, p < 0.01), the Malaysian data (β = 0.545, p < 0.01), and the Bangladeshi data (β = 0.314, p < 0.01). H2 was supported in the full data (β = 0.584, p < 0.01), the Malaysian data (β = 0.651, p < 0.01), and also in the Bangladeshi data (β = 0.350, p < 0.01). H3 was found to be supported in the full data (β = 0.567, p < 0.01), the Malaysian data (β = 0.471, p < 0.01) as well as in the Bangladeshi data (β = 0.545, p < 0.01).

*Hypotheses related to operating core (innovation process, cross*-*functional organisation, and implementation of tools/technology) and competition*-*informed pricing* The result of H4 is supported in the full data set (β = 0.170, p < 0.05) and the Malaysian data set (β = 0.255, p < 0.05), while in the Bangladeshi data it was not supported. The result of H5 was supported in the full data set (β = 0.266, p < 0.05) and the Bangladeshi data set (β = 0.275, p < 0.05), while in the Malaysian data set it was not supported. The result of H6 was supported in the full data set (β = 0.295, p < 0.01) and the Bangladeshi data set (β = 0.536, p < 0.01), while in the Malaysian data set, H6 was not supported.

*Hypotheses related to competition*-*informed pricing and performance* The findings revealed that H7 was supported in all the data sets, the full (β = 0.602, p < 0.01), the Malaysian (β = 0.562, p < 0.01), and the Bangladeshi (β = 0.596, p < 0.01) data sets.

*Hypotheses related to the mediating effect of competition*-*informed pricing on the relationship between operating core and performance*. The result shows that H9 was supported only in the full data set. H10 was supported in the full and in the Bangladeshi data sets only, but not in the Malaysian data set (Table 6).

**Table 6:** Result for mediating effect

	Path	Full sample (n = 176)				Malaysia sample (n = 98)				Bangladesh sample (n = 78)			
		SE	a*b	t value	Result	SE	a*b	t value	Result	SE	a*b	t value	Result
H8.	Process-COMIP-Performance	0.064	0.103	1.614	NS	0.083	0.143	1.732	NS	0.077	−0.006	−0.084	NS
H9.	Organisation-COMIP-Performance	0.075	0.160	2.128*	S	0.091	0.134	1.482	NS	0.092	0.164	1.772	NS
H10.	Tools/technology-COMIP-Performance	0.056	0.177	3.154**	S	0.068	0.092	1.350	NS	0.077	0.319	4.140**	S

*COMIP* competition-informed pricing, *OC* organisational culture

** p < 0.01, * p < 0.05

To explore the differences, we carried out PLS multi-group analysis for the Bangladeshi and Malaysian subsamples. We tested the differences between the path coefficients across the respective two data sets and the result is shown in Table 7. Three paths differ significantly between the two countries’ data sets. Organisational culture and process (p = 0.036); organisational culture and organisation (p = 0.003); tools or technology and competition-informed pricing (p = 0.016) have significant statistical differences (Table 7).

**Table 7:** Path differences by Country

	Bangladesh		Malaysia		Test for equality of variance	t value	p	Sig.
	Beta	SE	Beta	SE				
OC → Process	0.314	0.088	0.545	0.069	0.119	2.119	0.036	*
OC → Organisation	0.350	0.082	0.651	0.060	0.029	2.997	0.003	**
OC → Tools/technology	0.545	0.064	0.471	0.091	1.000	0.635	0.526	–
Process → COMIP	−0.011	0.126	0.255	0.138	0.970	1.394	0.165	–
Organisation → COMIP	0.275	0.144	0.239	0.151	0.934	0.170	0.865	–
Tools/technology → COMIP	0.536	0.100	0.163	0.112	0.980	2.433	0.016	**
COMIP → Performance	0.596	0.069	0.562	0.056	0.185	0.393	0.695	–

*COMIP* competition-informed pricing, *OC* organisational culture

** p < 0.01, * p < 0.05

## Discussion

The results of the study show significant relationship between organisational culture and operating core (innovation process, cross-functional organisation, and implementation of tools/technology) in both Bangladesh and Malaysia context. It is in line with the previous notion regarding the fact that internal behaviour and external relation, as part of organisational culture, facilitates the implementation of innovation successfully in the developed countries context (Naranjo-Valencia et al. 2011). Similar findings have been also observed in the current study, which focuses on developing countries. Organisational culture as a source of new ideas (Uzkurt et al. 2013) facilitates the practice of operating core (innovation process, cross-functional organisation, and implementation of tools/technology) in telecommunications industry. Earlier researchers found that training and championing have an influence on shaping up innovative organisations and processes (Hull and Tidd 2003a). However, the current study gives importance to the overall organisational culture in relationship with the practice of the operating core. Nevertheless, results of the present research also give such impression in the context of telecommunications sector in Malaysia and Bangladesh. It is not expected that such practice of organisational culture would be the same throughout all organisations or throughout all the countries. In line with similar considerations, the result of the multi-group analysis shows that the relationship between organisational culture and process as well as organisational culture and cross-functional organisation are significantly and statistically differ between Malaysian telecommunications industry and Bangladeshi telecommunications industry. Based on the findings, the practice of organisational culture in relationship with process (β = 0.545) and cross-functional organisation (β = 0.651) is stronger in the Malaysian telecommunications sector compared to the Bangladeshi telecommunications sector, where process holds a standard beta of 0.314 and cross-functional organisation accounts a standard beta of 0.350.

According to scholars, cultural differences have implications on the organisations where they are operating (Tayeb 1994). Furthermore it has been asserted that cultural values at individual or societal level are greatly influenced by the national culture (Thornton et al. 2011). National culture with low individualism accentuates on strong group solidity. The culture which possess the characteristics of uncertainty avoidance at higher level prefer to follow clear rules of conduct, while cultures low on uncertainty avoidance relish on novel events and value innovation. Cultures those are high on harmony focuses accepting matters as they are, and low level of harmony indicates the prominence of assertiveness to advance personal or group interests (Li et al. 2013). Therefore, in context of this study, it is the veritable fact that the organisational culture would differ between the companies of these countries, which might have been experienced due to the influence of different national culture. Perhaps, due to the advancement of modern and trending organisational culture in Malaysia, the telecommunication companies are able to blend mechanistic process and organic cross-functional organisation as practices of innovation on a concurrent basis. It can be argued that the multi-ethnicity setting of the Malaysian culture influences the organisational culture to practise both the mechanistic and organic structures simultaneously. In the Malaysian context, cooperativeness and steadiness has been entrenched in the society, which presumably are influenced by the cultural harmony of the nation. From an economic point of view, Malaysia is in the stage of development and is considered to be one of the emerging tigers of Asia. The government has already taken up various measures to achieve developed nation recognition and status. With this view, it is inferred that the culture of cooperativeness, creativity, efficacy, and competitiveness among the Malaysian telecommunication companies are supportive towards innovation driven in such a transitional stage. To be more specific, based on the data, the study believes that cooperativeness is one of the most significant dimensions of organisational culture followed by consistency, and innovativeness for the telecommunication companies of the both countries. Furthermore, among the Malaysian telecommunications companies, cooperativeness and consistency deemed to be carrying more weightage. On the other hand, cooperativeness and innovativeness are more important among the Bangladeshi telecommunications companies in order to shape up effective innovation practices.

The relationship between innovation process and competition-informed pricing is found to be significant in the Malaysian telecommunications sector whereas in the Bangladeshi telecommunications sector, it is insignificant. Theoretically, innovation process refers to the mechanistic stand of the organisation. According to Liker et al. (1999) and Tidd and Hull (2011), a mechanistic organisation is appropriate when the environment is efficient, effective, and stable. The findings of this study reflect what was advocated earlier in the context of innovation in developed countries. The Malaysian telecommunications sector is presumably at a mature stage with greater efficiency and effectiveness compared to the Bangladeshi telecommunications sector. Such an efficient and mature state of the industry instigates us to consider the most important stakeholder in the business environment such as competitors. With this contextual argument, it is noteworthy to state that the Malaysian telecommunications industry takes into account the competition-informed pricing practice with the mechanistic state of business operation. However, the innovation process can improve firm’s performance if the practice of gathering price related information from competitors is emphasized. Competition-informed pricing helps managers in the Malaysian telecommunications field to understand the upper-limit of the price decision while practising the innovation process for performance improvement. Therefore, it is important to mention that through the competition-informed pricing practice, the mechanistic state of organisation can assist to achieve performance.

In contrast to Malaysia, the relationship of cross-functional organisation and tools/technology with competition-informed pricing is significant in the Bangladeshi telecommunications sector. Bangladesh is in a position where it is about to take flight towards the development of innovation. Apparently, foreign investment is growing in the country, with greater interest among the telecommunication companies around the world. Therefore, the market is experiencing rapid changes in terms of organisational operation and strategy. As suggested by Liker et al. (1999) and Tidd and Hull (2011), organisations tend to be organic while the environment is not stable, dynamic, and the existence of less rules and regulations. In this scenario, it is justifiable to conclude upon the significance of the result that denotes the influence of cross-functional organisation on competitor-informed pricing. However, it is important to understand the competitors’ pricing strategy and competitors’ strength in the market through use of cross-functional team members within the innovative organisation. Computer information technology (CIT)’s tools, indeed, updates the process of service innovation cycle among cross-functional team members and increases the frequency of cross-functional team members’ communication in the value chain as highlighted in the previous study (Collins and Hull 2002; Tidd and Hull 2011). Thus, the result of the current study explains a facilitator role of competition-informed pricing for implementation of tools/technology to achieve a firm’s goals and performance only in the Bangladeshi telecommunications sector. Since the offered services of the telecommunications industry are very much similar across the companies, therefore, the state of competition is apparently higher, which triggers the companies to consider competition-informed pricing. In the result of multi-group analysis, the relationship between tools/technology and competition-informed pricing significantly differs in the Bangladeshi telecommunications sector (β = 0.536) compared to the Malaysian telecommunications sector (β = 0.163).

In line with the resource based view theory (RBV), organisation resources are converted to capabilities which would have an effect on competitive advantage (Barney 1991). In this study, resources namely innovation process, cross functional organisation, and tools/technology have causal effect on the firms capabilities that is competition-informed pricing. Subsequently, this capability (competition-informed pricing) has also a casual effect on competitive advantage, in this study which is performance. In this line, it has been argued in the literature that in capitalizing resources, an organisation can dominate and achieve a high level of performance (Barney 1991).

Interestingly, the mediating effect of competition-informed pricing is found to be significant on the relationship between tools/technology and performance only in the Bangladeshi data set. The reason probably accounts for the state of the progress in Bangladesh in terms of business innovation. Bangladesh is struggling towards the benchmark of the international standard. Being in transition from least developed country to emerging country, the business organisations are proactive to inculcate the practice of using tools/technology. On the other hand, tools/technology has become a part of the business operation for a fairly long time in Malaysia. Therefore, a significant difference has been observed between the Malaysian and Bangladeshi telecommunications sector in terms of these relationships.

## Managerial relevance

The illustrated research model is a useful theoretical framework for explaining the elements of operating core practices of service innovation that influence higher performance through the mediating effect of competition-informed pricing. According to the result attained from this study, managers of the Malaysian telecommunications sector do not take into account the competition-informed pricing while practising the operating core of service innovation to achieve higher performance. On the contrary, managers of the Bangladeshi telecommunication companies should take into account the competition-informed pricing while practising the operating core of service innovation to realize greater performance to counter the instable environment. The study also reflects the situation of organisational culture practice in both countries’ industry. It is recommended that managers of Bangladeshi telecommunications industry develop an organisational culture to gain performance advantages with the practice of service innovation.

Overall, the findings suggest that it is advantageous for the telecommunications industry to escalate the level of performance, facilitating managers to consider competition-pricing for new services with the support of operating core of the service innovation management. The managers of the industry must look towards competitors to set the price of the service along with practicing innovative process, innovative organisation, and implanting tools or technology. This may assist the managers to gain insight on the practice of service innovation, organisational culture, and performance.

## Conclusion

Taken all together, the results of this study show that the service innovation practice differs between Malaysia and Bangladesh. In Malaysia, organisational culture is revealed to be a strong predictor for operating core of service innovation compared to the Bangladeshi telecommunications sector. Furthermore, in the Malaysian telecommunications sector, competition-informed pricing does not necessitate playing any role between operating core of service innovation and performance, while in the Bangladeshi telecommunications sector, competition-informed pricing facilitates the relationship of tools or technology with performance. In addition, the relationship between tools or technology and competition-informed pricing is strong in the Bangladeshi telecommunications sector. On the other hand, it is not significant in the Malaysian telecommunications sector. It is however expected that if the respective managers of both countries consider these issues, it would contribute immensely towards the practice of service innovation management as a whole.

## Limitations and future directions of research

This paper has limitations that are to be noted. The paper is based on a single industry and the sample is drawn only from the telecommunications industry, which has the potential for limiting the generalisation of the findings of this research across other industries. This can be overcome by extending the scope of the research by using a larger database comprising responses of managers representing a number of industries. Although this paper is based purely on quantitative methodology using established constructs, these were not used in any prior study in Bangladesh and Malaysia. Future study can be developed using a mixed methodology comprising qualitative and quantitative approaches toward contributing to greater generalisation of the findings. In addition, future study can look into the other subsidies of Telenor group and Axiata group operating in Asian countries such as India, Pakistan, Myanmar, Indonesia, Brunei, and Thailand in order to test the applicability of the framework in the developing countries.

## Appendix

See Table 8.

**Table 8:** Measurement items

Variables	Measurements	Reference
Organisational culture	1. Managers treat all staff as their big family members 2. Employees are loyal to one another 3. Managers actively lead the staff to grow and innovate 4. Employees always have to face challenges which make them to learn and grow 5. Managers set up clear goals and ask employees to carryout goals strictly 6. Firm is stable and offers job security to employees 7. Managers emphasize working efficiency and acts effectively 8. Every department must compete with its peer for better efficiency 9. Every employee must compete with its peer for better efficiency	Chang and Lin (2007)
Process	1. Setting standards for the performance of services 2. Mapping processes to reduce non-value activities 3. Improving documentation of processes 4. Measuring conformance with processes 5. Institutionalizing continuous improvement processes	Hull (2003) and Hull and Tidd (2003a)
Cross-functional organisation	1. Cross-functional teaming 2. Cross-training specialists 3. Strengthening the role of project managers 4. Increasing the influence of downstream functions in upstream decisions, e.g. customer service input in product development 5. Reorganisation of jobs to reduce hand-offs	Hull (2003) and Hull and Tidd (2003a)
CIT tools	1. Internal communications via any computer networks e.g. e-mail 2. Updated information technology systems 3. Distributed databases online to multiple functions 4. Common software for process mapping 5. Built online databases with lessons learned and best-practice templates	Hull (2003) and Hull and Tidd (2003a)
Competition-informed pricing	1. The competitor’s current price strategy 2. The estimation of competitor’s strength to react 3. The market structure (number and strength of competitors) 4. The degree of competition on the market 5. The competitive advantages of competitors on the market	Ingenbleek et al. (2003)
Performance	1. Upgraded features 2. Higher quality 3. Easier customer use after purchase 4. Shorter time from concept to full-scale delivery of the service 5. Reduced cost of service development 6. Shorter response time to order for existing services 7. Shorter time for adjustments to complaints 8. Reduced cost of service delivery 9. Higher quality of delivery process, e.g. fewer customer complaints 10. Conformance with service development process and procedures	Hull and Tidd (2003a)

## References

[CR1] Ardichvili A, Maurer M, Li W, Wentling T, Stuedemann R (2006). Cultural influences on knowledge sharing through online communities of practice. J Knowl Manag.

[CR2] Aycan Z, Kanungo R, Mendonca M, Yu K, Deller J, Stahl G, Kurshid A (2000). Impact of culture on human resource management practices: a 10-country comparison. Appl Psychol.

[CR3] Barney JB (1986). Organisational culture: can it be a source of sustained competitive advantage?. Acad Manage Rev.

[CR4] Barney JB (1991). Firm resources and sustained competitive advantage. J Manage.

[CR5] Barrett M, Davidson E, Prabhu J, Vargo SL (2015). Service innovation in the digital age: key contributions and future directions. MIS Q.

[CR6] Blindenbach-Driessen F (2015). The (In) effectiveness of cross-functional innovation teams: the moderating role of organisational context. IEEE Trans Eng Manage.

[CR7] Büschgens T, Bausch A, Balkin DB (2013). Organisational culture and innovation: a meta-analytic review. J Prod Innov Manage.

[CR8] Chang SE, Lin C-S (2007). Exploring organisational culture for information security management. Ind Manage Data Syst.

[CR9] Chen C-J, Hsiao Y-C (2013). The endogenous role of location choice in product innovations. J World Bus.

[CR10] Chin WW, Marcoulides GA (1998). The partial least squares approach for structural equation modeling. Modern methods for business research.

[CR11] Chin WW, Vinzi VE, Chin WW, Henseler J, Wang H (2010). How to write up and report PLS analyses. Handbook of partial least squares.

[CR12] Cohen J (1988). Statistical power analysis for the behavioral sciencies.

[CR13] Collins PD, Hull FM (2002). Early simultaneous influence of manufacturing across stages of the product development process: impact on time and cost. Int J Innov Manag.

[CR14] Damanpour F (2014). Footnotes to research on management innovation. Org Stud.

[CR15] De Jong JPJ, Vermeulen PAM (2003). Organizing successful new service development: a literature review. Manag Decis.

[CR16] Dutta S, Zbaracki MJ, Bergen M (2003). Pricing process as a capability: a resource-based perspective. Strateg Manag J.

[CR17] Efron B (1979). Bootstrap methods: another look at the jackknife. Ann Stat.

[CR18] Eisingerich AB, Rubera G, Seifert M (2009). Managing service innovation and interorganisational relationships for firm performance; to commit or diversify?. J Serv Res.

[CR19] Erumban AA, De Jong SB (2006) Cross-country differences in ICT adoption: a consequence of culture? J World Bus 41(4):302–314

[CR20] Fornell C, Larcker DF (1981). Evaluating structural equation models with unobservable variables and measurement error. J Mark Res.

[CR21] Goto A (2009). Innovation and competition policy*. Jpn Econ Rev.

[CR22] Götz O, Liehr-Gobbers K, Krafft M, Vinzi VE, Chin WW, Henseler J, Wang H (2010). Evaluation of structural equation models using the partial least squares (PLS) approach. Handbook of partial least squares.

[CR23] Hair JF, Hult GTM, Ringle C, Sarstedt M (2013). A primer on partial least squares structural equation modeling (PLS-SEM).

[CR24] Hair JF, Black WC, Babin BJ, Anderson RE (2009) Multivariate data analysis. Pearson Prentice Hall, Upper Saddle, New Jersey

[CR25] Hair JF, Ringle CM, Sarstedt M (2011). PLS-SEM: indeed a silver bullet. J Market Theory Prac.

[CR26] Henseler J, Ringle C, Sinkovics R (2009). The use of partial least squares path modeling in international marketing. Adv Int Market (AIM).

[CR27] Hinterhuber A (2004). Towards value-based pricing—an integrative framework for decision making. Ind Mark Manage.

[CR28] Hogan SJ, Coote LV (2014). Organisational culture, innovation, and performance: a test of Schein’s model. J Bus Res.

[CR29] Hull FM, Tidd J, Hull FM (2003). Product development in service enterprises: Case studies of good practice. Service innovation: organisational responses to technological opportunities and market imperatives.

[CR30] Hull FM (2004). Innovation strategy and the impact of a composite model of service product development on performance. J Serv Res.

[CR31] Hull FM, Tidd J, Tidd J, Hull FM (2003). A composite framework of product development and delivery effectiveness in services. Service innovation; organisation responses to technological oOpportunities and market imperatives.

[CR32] Hull FM, Tidd J, Tidd J, Hull F (2003). The organisation of new service development in the USA and UK. Service innovation; organisation responses to technological opportunities and market imperatives.

[CR33] Hull FM, Collins PD, Liker JK (1996). Composite forms of organisation as a strategy for concurrent engineering effectiveness. IEEE Trans Eng Manage.

[CR34] Hultink EJ, Griffin A, Hart S, Robben HS (1997). Industrial new product launch strategies and product development performance. J Prod Innov Manage.

[CR35] Ingenbleek P, Debruyne M, Frambach RT, Verhallen TMM (2003). Successful new product pricing practices: a contingency approach. Market Lett.

[CR36] Iorgulescu A, Marcu M (2015). The relationship between national culture and organisational culture. Soc Sci Educ Res Rev.

[CR37] Jiménez-Jiménez D, Sanz-Valle R (2011). Innovation, organisational learning, and performance. J Bus Res.

[CR38] Klein EE, Dologite DG (2000). The role of computer support tools and gender composition in innovative information system idea generation by small groups. Comput Hum Behav.

[CR39] Lee S-G, Trimi S, Kim C (2013). The impact of cultural differences on technology adoption. J World Bus.

[CR40] Li K, Griffin D, Yue H, Zhao L (2013). How does culture influence corporate risk-taking?. J Corp Finance.

[CR41] Liker JK, Collins PD, Hull FM (1999). Flexibility and standardization: test of a contingency model of product design—manufacturing integration. J Prod Innov Manage.

[CR42] Magnusson PR, Matthing J, Kristensson P (2003). Managing user involvement in service innovation experiments with innovating end users. J Serv Res.

[CR43] Malaysian Investment Development Authority (2014) Services sector. http://www.mida.gov.my/home/services-sector/posts/

[CR44] Martins E, Terblanche F (2003). Building organisational culture that stimulates creativity and innovation. Eur J Innov Manage.

[CR45] McConnell C, Brue S, Flynn S (2009). Economics: principles, problems, and policies.

[CR46] Miron E, Erez M, Naveh E (2004). Do personal characteristics and cultural values that promote innovation, quality, and efficiency compete or complement each other?. J Org Behav.

[CR47] Mudrak T, van Wagenberg A, Wubben E (2005). Innovation process and innovativeness of facility management organisations. Facilities.

[CR48] Naranjo-Valencia JC, Jiménez-Jiménez D, Sanz-Valle R (2011). Innovation or imitation? The role of organisational culture. Manag Decis.

[CR49] Noam EM (2006). Fundamental instability: why telecom is becoming a cyclical and oligopolistic industry. Inf Econ Policy.

[CR50] OECD (2007) Innovation and growth-rationale for an innovation strategy. http://www.oecd.org/science/inno/39374789.pdf

[CR51] Orfila-Sintes F, Crespí-Cladera R, Martínez-Ros E (2005). Innovation activity in the hotel industry: evidence from Balearic Islands. Tour Manag.

[CR52] Ottenbacher MC (2007). Innovation management in the hospitality industry: different strategies for achieving success. J Hosp Tour Res.

[CR53] Planning Commission (2012) Perspective plan of Bangladesh 2010––2021, general economics division planning commission government of the People’s Republic of Bangladesh. http://www.plancomm.gov.bd/wp…/09/Perspective-Plan-of-Bangladesh.pdf

[CR54] Podsakoff PM, MacKenzie SB, Lee J-Y, Podsakoff NP (2003). Common method biases in behavioral research: a critical review of the literature and recommended remedies. J Appl Psychol.

[CR55] Ringle C, Wende W (2005) SmartPLS. http://www.smartpls.de

[CR56] Shapiro BP, Jackson BB (1978). Industrial pricing to meet customer needs. Harv Bus Rev.

[CR57] Taghizadeh SK, Jayaraman K, Rahman SA, Malkifar S (2014). A glance on service innovation scenario: case of leading telecommunication companies in Malaysia. Int J Bus Innov.

[CR58] Tayeb M (1994). Organisations and national culture: methodology considered. Org Stud.

[CR59] Thornton PH, Ribeiro-Soriano D, Urbano D (2011). Socio-cultural factors and entrepreneurial activity: an overview. Int Small Bus J.

[CR60] Tidd J, Bessant J (2009). Managing innovation: integrating technological, market and organisational change.

[CR61] Tidd J, Hull FM (2011). Service innovation: development, delivery and performance. The handbook of innovation and services: a multi-disciplinary perspective.

[CR62] Tidd J, Bessant J, Pavitt K (2005). Managing innovation: integrating technological, market and organisational change.

[CR63] Uzkurt C, Kumar R, Kimzan HS, Eminoglu G (2013). Role of innovation in the relationship between organisational culture and firm performance: a study of the banking sector in Turkey. Eur J Innov Manage.

[CR64] van Riel ACR (2005). Introduction to the special issue on service innovation management. Manag Serv Qual.

[CR65] Vermeulen P, van der Aa W, Tidd J, Hull FM (2003). Organizing innovation in services. Service innovation, organizational responses to technological opportunities and market imperatives.

[CR66] Weiss DS, Legrand C (2011). Innovative intelligence: the art and practice of leading sustainable innovation in your organisation.

[CR67] World Economic Forum (2015) The global competitiveness report 2014–2015. In: Schwab (ed). Geneva. Available via http://www3.weforum.org/docs/WEF_GlobalCompetitivenessReport_2014-15.pdf. Accessed 21 Sept 2015

[CR68] Yung YF, Bentler PM (1994). Bootstrap-corrected ADF test statistics in covariance structure analysis. Br J Math Stat Psychol.

[CR69] Zammuto RF, O’Connor EJ (1992). Gaining advanced manufacturing technologies’ benefits: the roles of organisation design and culture. Acad Manag Rev.

